# Hands-on time during cardiopulmonary resuscitation is affected by the process of teambuilding: a prospective randomised simulator-based trial

**DOI:** 10.1186/1471-227X-9-3

**Published:** 2009-02-14

**Authors:** Sabina Hunziker, Franziska Tschan, Norbert K Semmer, Roger Zobrist, Martin Spychiger, Marc Breuer, Patrick R Hunziker, Stephan C Marsch

**Affiliations:** 1 Medical Intensive Care Unit, University of Basel, 4031 Basel, Switzerland; 2 Department of Psychology, University of Neuchâtel, 2000 Neuchâtel, Switzerland; 3 Department of Psychology, University of Bern, 3000 Bern, Switzerland; 4 Didavis Center for Medical Education and Simulation, 4103 Bottmingen, Switzerland

## Abstract

**Background:**

Cardiac arrests are handled by teams rather than by individual health-care workers. Recent investigations demonstrate that adherence to CPR guidelines can be less than optimal, that deviations from treatment algorithms are associated with lower survival rates, and that deficits in performance are associated with shortcomings in the process of team-building. The aim of this study was to explore and quantify the effects of ad-hoc team-building on the adherence to the algorithms of CPR among two types of physicians that play an important role as first responders during CPR: general practitioners and hospital physicians.

**Methods:**

To unmask team-building this prospective randomised study compared the performance of preformed teams, i.e. teams that had undergone their process of team-building prior to the onset of a cardiac arrest, with that of teams that had to form ad-hoc during the cardiac arrest. 50 teams consisting of three general practitioners each and 50 teams consisting of three hospital physicians each, were randomised to two different versions of a simulated witnessed cardiac arrest: the arrest occurred either in the presence of only one physician while the remaining two physicians were summoned to help ("ad-hoc"), or it occurred in the presence of all three physicians ("preformed"). All scenarios were videotaped and performance was analysed post-hoc by two independent observers.

**Results:**

Compared to preformed teams, ad-hoc forming teams had less hands-on time during the first 180 seconds of the arrest (93 ± 37 vs. 124 ± 33 sec, P < 0.0001), delayed their first defibrillation (67 ± 42 vs. 107 ± 46 sec, P < 0.0001), and made less leadership statements (15 ± 5 vs. 21 ± 6, P < 0.0001).

**Conclusion:**

Hands-on time and time to defibrillation, two performance markers of CPR with a proven relevance for medical outcome, are negatively affected by shortcomings in the process of ad-hoc team-building and particularly deficits in leadership. Team-building has thus to be regarded as an additional task imposed on teams forming ad-hoc during CPR. All physicians should be aware that early structuring of the own team is a prerequisite for timely and effective execution of CPR.

## Background

Strict adherence to internationally accepted guidelines for cardiopulmonary resuscitation (CPR) [1-4] is a prerequisite to improve survival rates in cardiac arrest [5-7]. Still, outcome after CPR has remained disappointingly poor for decades. Thus, there is an unmet need to optimise the performance of CPR in daily life.

Cardiac arrests are handled by teams rather than by a single individual. Usually, these teams form ad-hoc during the event as different health-care workers join the first person present. Thus, in cardiac arrests, physicians have the dual task of building an efficient team and provide patient's support simultaneously. Recent investigations demonstrate that adherence to CPR guidelines can be less than optimal [8-15], that deviations from treatment algorithms are associated with lower survival rates [14], and that deficits in performance were associated with shortcomings in the process of team-building [11,13]. Thus, improvements in the process of team-building could be a key factor for increasing the quality and hence the success rates of CPR.

There are significant practical and ethical problems in investigating team-related issues like team-building during CPR in real cases: 1) cardiac arrests are emergencies and not planned events; 2) as team-building occurs during the early phase of a cardiac arrest, trained observers had to be at the scene from the very onset of the arrest, which under most circumstances is impractical to achieve; and 3) for ethical reasons such observers had to intervene immediately in case of obvious deficits in CPR which would invalidate the use of their observational data for the investigation of the teams' performance.

Medical simulation allows the investigation of issues that for a variety of medical, practical, and ethical reasons are difficult, if not impossible to investigate in real patients. As far as CPR is concerned, simulation allows planned and repetitive investigations with perfectly identical conditions for all participants. Moreover, simulation allows recording of objective data from both "patients" and physicians right from the start of a simulated cardiac arrest and, therefore, appears to be perfectly suited to investigate the process of team-building during CPR.

The aim of the present study was to explore and quantify the effects of the process of ad-hoc team-building on the adherence to algorithm in CPR. We studied two types of physicians that both play an important role as first responders during CPR: general practitioners and hospital physicians. To unmask team-building we compared the performance of preformed teams, i.e. teams that had undergone their process of team-building prior to the onset of a cardiac arrest with that of teams that had to form ad-hoc during the cardiac arrest.

## Methods

### Participants

The study took place between 2002 and 2005 during consecutive workshops at the simulation centre of the University of Basel, Basel, Switzerland. The workshops were marketed as "unique learning experience of relevant medical emergencies in a patient simulator" and physicians could take part on a voluntary basis. No formal previous training was required to participate and, during the workshop, no training or teaching was provided *prior *to the simulation. Thus, the participants' performance reflected their current knowledge and skills. Participants were general practitioners involved in emergency duties or hospital physicians of different specialities (internal medicine, cardiology, intensive care) and status (staff physicians, residents, juniors). The study was approved by the local ethical committee and written informed consent was obtained from all participants.

### Simulator

A high-fidelity patient simulator (Human Patient Simulator, METI^®^, Sarasota, FL, USA) was used. Features of this simulator include palpable pulses, spontaneous breathing with visible thoracic excursion, eyes with spontaneous lid movements, and a speaker in the mannequin's head that broadcasts the voice of an operator to give the illusion that the "patient" can talk. However, the simulator is unable to detect and/or record the depth of chest compressions and the adequacy of mask ventilation. A cannula was placed in a peripheral vein to allow for intravenous administration of drugs. A commercially available manual defibrillator was placed next to the bed. All participants received a 15 min structured instruction on the technicalities of the simulator.

### Study design

This is a prospective randomized study. Each resuscitation team consisted of a nurse and either three general practitioners or three hospital physicians. The nurse belonged to the simulator team and was instructed to display a helpful attitude, but to be active on commands only.

Using sealed envelopes a stratified randomization according to the participants' profession was employed to assign an equal number of teams composed of either general practitioners or hospital physicians to two different versions of a scenario of a simulated witnessed cardiac arrest: version "ad-hoc" mimics reality in that only one physician, randomly selected from his/her team, was present at the start of the scenario and the remaining two physicians were summoned to help upon the onset of the cardiac arrest; in version "preformed" all three physicians were present right from the start of the scenario. Pilot experiments revealed that a time period of approximately 5 min during which preformed teams together receive information about the patient's history and subsequently assess together the patient is sufficient to structure the team, and that longer time periods feasible within the settings of simulation offer no significant advantage.

### Scenario

Prior to the simulation, teams were instructed that they were the responsible physicians for the "patient" and that a nurse, fully familiar with all technicalities of the simulator and the equipment, would help them upon request. Teams of general practitioners were informed that the scenario would take place in a group practice where all three of them would work. Teams of hospital physicians were informed that the scenario would take place in the ambulatory part of a hospital where all three of them would work. In "ad-hoc" teams, two randomly selected members were then led to a room adjacent to the simulator and the remaining physician was instructed that help from his/her colleagues would be immediately available on request. Thereafter, the case history was given to the one remaining physician of the "ad-hoc" teams or to all three physicians of the "preformed" teams.

The "patient" was a 66 year old man who felt dizzy after an uneventful bicycle stress test. Upon entering the simulator room, the physician(s) encountered a talkative "patient" connected to a monitor showing sinus rhythm. The "patient" did not feel dizzy anymore but volunteered a detailed account of that episode. In addition, the "patient" complained of stiff muscles in both thighs. Two minutes after the physician(s) had entered the simulator, a cardiac arrest occurred due to ventricular tachycardia displayed on the monitor. With the onset of the cardiac arrest, the "patient" closed his eyes, ceased to speak and to breathe, and pulses were no longer palpable. As our aim was to study the effects of team-building during the early phase of a cardiac arrest, we ensured that all ad-hoc teams were complete ≤ 20 sec after the start of the cardiac arrest: in case the first physician of the "ad-hoc" group did not call for his colleagues within 15 sec they were immediately sent to the simulator. Regardless of any measures taken the patient stayed in cardiac arrest for 3 min. Thereafter, sinus rhythm could be achieved by defibrillation. To avoid a potentially traumatic experience the death of the "patient" was prevented by the nurse who, after six minutes, suggested appropriate measures.

Upon completion of the scenario participants were given a questionnaire and asked to rate on a 11-point Likert scale [16] the realism of the scenario, the realism of their own behaviour, and the realism of the behaviour of their colleagues (0 = "not at all realistic", 5 = "somewhat realistic", 10 = "very realistic"); the quality of their team's performance (0 = "very low performance", 5 = "average performance", 10 = "very high performance"); the stress felt during simulation, and the stress felt during a real cardiac arrest (0 = "no stress at all felt", 5 = "some stress felt", 10 = "very high stress felt"). A video-assisted debriefing concluded the simulation.

### Data analysis

Using frame-in-frame technology, the teams' performance and the monitor displaying the "patient's" vital signs were simultaneously recorded. Data were coded based on the video-tapes recorded during simulation by two independent observers. Inter-observer agreement was assumed if the difference of timing of events was less than 5 sec. In this case, the shorter of two different timings was used for further analysis. Disagreements of more than five seconds in the timing of events were solved by jointly reviewing the videotapes.

Hands-on time was defined as cardiac massage or defibrillation. Each defibrillation was rated as 10 sec of hands-on time. Interruptions of cardiac massage to allow for ventilation were rated as continuous cardiac massage if the interruption was ≤ 10 sec. The first appropriate intervention was defined as first execution of either precordial thump, ventilation, cardiac massage, or defibrillation. Chest compression rates were calculated during the third minute after the onset of the cardiac arrest using a previously published formula [8]: compression rate = (compressions per 60-second segment) × 60/(60 – total pause time in the 60-second segment), where pause time indicates periods of time in which ≥ 2 seconds pass without chest compressions.

All utterances during the first 3 min after the cardiac arrest were noted and classified according to a predefined checklist partly based on the adapted Leadership Behaviour Description Questionnaire [17]: *Decision what should be done *was defined as any utterance, regardless whether correct or followed, on measures to be performed (e.g. we should defibrillate); *Decision on how things should be done *was defined as any utterance, regardless whether correct or followed, on how to perform a measure (e.g. the next countershock should be performed with 360 Joule); *Direction/Command *was defined as any utterance, regardless whether correct or followed, prompting a colleague to do something or do it differently (e.g. you should perform the massage quicker); *Task assignment *was defined as any utterance, regardless whether correct or followed, that assigned a team member to a particular task. *Reflection *was defined as any utterance, regardless whether correct or followed, with the potential of prompting a colleague or the team to assess the situation (e.g. what should we do next?). *Other utterance *was defined as any utterances that did not fit in one of the above categories.

### Statistics

The primary outcome was the hands-on time during the first three minutes of the cardiac arrest. Secondary outcomes included the timing of measures of resuscitation and leadership utterances. A difference of ≥ 10% (i.e. a difference ≥ 18 sec in the first 180 sec of the arrest) in the primary outcome hands-on time was considered to be of clinical significance. Interruptions of cardiac massage of this magnitude are associated with poorer survival rate and worse neurological outcomes [18,19]. A power analysis revealed that 45 teams had to be studied in each group to detect this difference with significance levels of 0.05 and 90% power. Anticipating a 10% rate of technical difficulties or major protocol deviations we planed to include 50 teams of general physicians and 50 teams of hospital physicians in the study. Data were analysed using SPSS (version 15.0), a commercially available statistical software. Cohen's Kappa for inter-rater reliability, general linear modelling, stepwise multiple linear regression, and Student's t-test were used as appropriate. A p < 0.05 was considered to represent statistical significance.

## Results

### Enrolment and analysis

150 general practitioners and 150 hospital physicians were allocated to 100 teams, composed of either three general practitioners or three hospital physicians. All 300 physicians participated only once, all 100 teams were randomised and completed the simulated scenario as intended, and no protocol violations occurred. Due to an incomplete video recording, one team (hospital physicians, version preformed teams) had to be excluded from the analysis. Thus, data of 99 teams were analysed [see Additional file 1 for CONSORT flowchart of the study]. Demographics of the participants are displayed in table 1.

**Table 1:** Demographics of participants

	General Practitioners		Hospital Physicians	
	Preformed (n = 75)	Ad-hoc (n = 75)	Preformed (n = 75)	Ad-hoc (n = 75)

Age (years)	47 (4)	49 (5)	42 (4)*	41 (3)*

Sex (f/m)	19/56	14/61	28/47	26/49

Position (staff/resident/junior)			25/46/4	33/32/10

Speciality (FM/IM/Card/CC)	51/22/3/0	48/21/6/0	0/42/21/12	0/39/18/18

Means (SD)

* P < 0.01 vs. general practitioners with same scenario.

FM = family medicine; IM = internal medicine; Card = Cardiology; CC = critical care

There was no inter-rater disagreement for the timing of events. The inter-rater agreement for the classification of utterances was very high (Cohen's Kappa 0.98; p ≤ 0.007); all disagreements were solved by jointly reviewing the video recordings.

Two teams of general physicians (one of each version of the scenario) did not complete the scenario despite of suggestions by the nurse: one team did not perform cardiac massage at all and the other team performed no further defibrillation after their second countershock.

### Primary outcome

**Figure 1:**
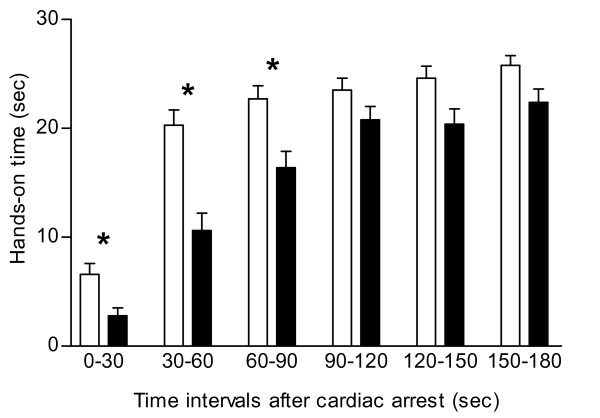
**Hands-on time in witnessed cardiac arrests**. Hands-on time during consecutive 30 sec intervals during the first 180 sec after the onset of a witnessed cardiac arrest. Data are means ± SEM; open bars = preformed teams; filled bars = ad-hoc forming teams; * = P < 0.001 for difference between the type of teams during time interval indicated. As hands-on times did not differ between general practitioners and hospital physicians, for the sake of clarity bars were not further subdivided according to type of physician.

Ad-hoc teams had significantly shorter hands-on times during the first 3 min of the cardiac arrest than preformed teams (table 2, figure 1). General practitioners and hospital physicians did not differ in the hands-on time (108 ± 37 sec vs. 110 ± 34 sec).

**Table 2:** Timing of resuscitation measures after the onset of cardiac arrest

		All	General practitioners	Hospital physicians
Hands-on time during the first 180 sec	Preformed (n = 49)	124 (33)	121 (36)	127 (31)

	Ad-hoc (n = 50)	93 (37) *	96 (38)^¶^	90 (36)^†^

				

First appropriate intervention (sec)	Preformed (n = 49)	24 (16)	24 (17)	24 (16)

	Ad-hoc (n = 50)	43 (28)*	45 (35)^†^	40 (21)^†^

				

First defibrillation (sec)	Preformed (n = 49)	67 (42)	84 (46)	51 (30)^§^

	Ad-hoc (n = 50)	107 (46)*	113 (47)^†^	101 (45)*

				

Start of cardiac massage (sec)	Preformed (n = 49)	60 (48)	58 (47)	61 (50)

	Ad-hoc (n = 50)	76 (57)	71 (64)	82 (49)

				

Chest compression rate (comp/min)	Preformed (n = 49)	82 (22)	75 (21)	88 (21)^§^

	Ad-hoc (n = 50)	85 (16)	79 (17)	91 (12)^§^

				

Administration of Epinephrine (sec)	Preformed (n = 49)	157 (55)	168 (56)	148 (54)

	Ad-hoc (n = 50)	210 (70)*	230 (76)^†^	190 (58)^†^^§^

Means (SD)

* = p < 0.0001 vs. preformed teams

^† ^= p < 0.01 vs. preformed teams

^¶ ^= p < 0.05 vs. preformed teams

§ = p < 0.05 vs. general practitioner in same scenario.

### Secondary outcomes

The first appropriate interventions were precordial thump (28 of 99 teams), cardiac massage (28), ventilation (24), and defibrillation (19), respectively with no statistically significant differences between types of physicians and team type. Seven teams (6 general practitioners) never administered epinephrine (p = 0.11 for general practitioners vs. hospital physicians); and seven teams (all hospital physicians) administered an anti-arrhythmic drug prior to the administration of epinephrine (p = 0.006 for hospital physicians vs. general practitioners).

**Figure 2:**
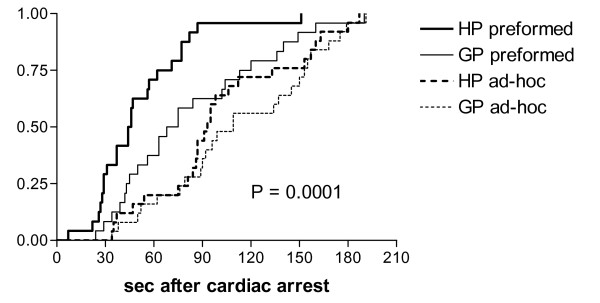
**Timing of defibrillation**. Survival curve of the timing of the first defibrillation in simulated witnessed cardiac arrest. Time 0 denotes the onset of cardiac arrest. HP = teams composed of 3 hospital physicians and one nurse; GP = teams composed of 3 general practitioners and one nurse; preformed = witnessed arrest occurring in the presence of the complete team; ad-hoc = witnessed arrest occurring in the presence of one physician and one nurse and the remaining two physicians are summoned to help.

Ad-hoc teams performed the first appropriate intervention, the first defibrillation, and the administration of epinephrine significantly later than preformed teams (table 2, figure 2). Compression rates below recommendations of = 80/min [3] were observed in 20 preformed (10 general practitioners and 10 hospital physicians) and 15 ad-hoc teams (12 general practitioners and 3 hospital physicians) resulting in p = 0.4 for preformed vs. ad-hoc teams and p = 0.09 for general practitioners vs. hospital physicians. General practitioners performed defibrillation (98 ± 48 vs 77 ± 46 sec, p = 0.023) and administered epinephrine (201 ± 74 vs 169 ± 60 sec, p = 0.021) later than hospital physicians and had lower compression rates (77 ± 19 vs 90 ± 17. compressions/min, p = 0.001) (table 2).

In ad-hoc teams we observed less leadership utterances but more reflection than in preformed teams (table 3). There was no significant difference between general practitioners and hospital physicians for the number and type of utterances. When type of team, type of physician, direction/command, task assignment, and decision how where entered in a stepwise multiple regression with the primary outcome (hands-on time during the first 3 min) as dependent variable only direction/command remained in the equation, the parameter estimate being + 4.6 sec support/utterance (r^2 ^= 0.16; P < 0.0001).

**Table 3:** Classification of utterances occurring during the first 3 min after the onset of cardiac arrest

		All	General practitioners	Hospital physicians
All utterances	Preformed (n = 49)	43.8 (10.9)	44.6 (12.2)	43.0 (9.6)

	Ad-hoc (n = 50)	42.6 (11.5)	44.4 (9.8)	40.9 (13.0)

				

Leadership utterances	Preformed (n = 49)	19.8 (5.7)	19.0 (5.7)	20.5 (5.6)

	Ad-hoc (n = 50)	13.9 (4.7)*	13.1 (4.9)*	14.6 (4.5)*

				

Direction/command	Preformed (n = 49)	6.8 (3.4)	6.7 (3.0)	6.9 (3.9)

	Ad-hoc (n = 50)	3.8 (2.4)*	3.8 (2.6)*	3.7 (2.2)*

				

Decision what	Preformed (n = 49)	7.2 (2.3)	7.0 (2.3)	7.4 (2.2)

	Ad-hoc (n = 50)	6.6 (2.4)	5.9 (2.4)	7.3 (2.4)

				

Decision how	Preformed (n = 49)	4.0 (2.2)	3.7 (2.6)	4.3 (1.7)

	Ad-hoc (n = 50)	2.8 (1.7)*	2.5 (1.9)	3.0 (1.6)^†^

				

Task assignment	Preformed (n = 49)	1.7 (1.5)	1.6 (1.6)	1.8 (1.3)

	Ad-hoc (n = 50)	0.7 (0.8)*	0.9 (0.8)	0.6 (0.8)*

				

Reflection	Preformed (n = 49)	5.4 (3.7)	6.6 (4.1)	4.1 (2.7)^§^

	Ad-hoc (n = 50)	7.5 (3.8)*	7.7 (3.9)	7.3 (3.7)*

				

Other utterances	Preformed (n = 49)	17.4 (6.4)	18.0 (6.9)	16.8 (6.1)

	Ad-hoc (n = 50)	20.7 (8.5)^†^	22.8 (7.5)^†^	18.6 (8.9)

Means (SD); * = p < 0.01 vs. preformed teams; † = p < 0.05 vs. preformed teams; § = P < 0.05 vs. general practitioner in same scenario

The median participants' ratings were 9 (Inter-quartile-range [IQR] 8 – 10) for the realism of the scenario, 8 (IQR 8 – 10) for the realism of their own behaviour, 8 (IQR 7 – 10) for the realism of the behaviour of their colleagues, 7 (IQR 5 – 10) for the quality of their team's performance, 6 (IQR 4 – 10) for the stress felt during simulation, and 9 (IQR 7 – 10; p < 0.0001 vs. stress during simulation) for the stress felt during a real cardiac arrest. None of the above ratings was significantly affected by study group, profession, or objective performance measures.

## Discussion

Teams that have to form ad-hoc during a cardiac arrest provide 30 sec less hands-on time during the initial 3 min and delay the first defibrillation by 40 sec when compared to teams that had the opportunity to form prior to the cardiac arrest.

Our findings support the growing awareness of a less than optimal adherence to algorithms of CPR [8-14] which partly explains the poor outcome of cardiac arrests [14,20]. Considering the optimal starting conditions (witnessed cardiac arrest in a monitored patient, presence of at least one physician and a nurse, defibrillator available at bedside), the performance of many teams was surprisingly poor regardless whether general practitioners or hospital physicians were involved. If we grant the teams an initial 20 sec for diagnosis and to organise themselves, the hands-off times of the preformed teams during the initial 3 min of the arrest were on average 40 sec (i.e. more than 20% of the time available) while the hands-off times of the ad-hoc teams amounted to 70 sec (i.e. almost 40% of the time available).

Immediate defibrillation is a class I recommendation in a witnessed cardiac arrest. Similar to previous work [11,14,21] we observed unnecessary delays in the time to defibrillation. According to recent registry data, a delay in defibrillation of more than 2 min occurs in 30% of in-hospital arrests [14]. In the present study 36% (18 out of 50) of the ad-hoc forming teams, but only 12% (6 out of 49) of the preformed teams delayed their first countershock beyond 2 min. Thus, in addition to patient and hospital related variables identified by previous work [14] team related issues are important factors to explain delays in the time to defibrillation.

Even if dedicated emergency teams exist within a community or institution, such teams are usually not immediately available at the onset of a cardiac arrest. Thus, as a clinical reality most if not all medical emergencies have to be handled, at least initially, by first responders in ad-hoc forming teams. Accordingly, we selected to study the initial 3 min of a cardiac arrest. Regardless whether general practitioners or hospital physicians were involved, our data demonstrate shortcomings in the quality rather than the quantity of communication during the early phase of CPR in ad-hoc forming teams: despite an equal number of total utterances, ad-hoc teams made significantly less leadership utterances. Structuring leadership of both team and task has been found to positively correlate with effective team performance during CPR [17,22]. Our findings demonstrate that the process of structuring the own team during the early phases of a medical emergency has to regarded as an important additional task. Deficiencies in this process, and particularly shortcomings in leadership behaviour, can result in significant delays in life-saving measures and deviations from treatment algorithms.

To the best of our knowledge, this is the first head-to-head comparison of the performance and team-building abilities of general practitioners and hospital physicians in a medical emergency. In emergencies occurring in the community or their practice general practitioners are acting as first responders and their performance is thus of outmost importance [23-27]. Surveys suggest that general practitioners are inadequately equipped and are not fully familiar with the current guidelines for optimal CPR performance [23,25,28]. By contrast, a recent analysis of self-reports revealed that adequately equipped general practitioners following the algorithms of CPR can achieve remarkable survival rates [27]. In the present study, general practitioners defibrillated later and administered epinephrine later than hospital physicians. In accordance with the literature, this may be related to the less frequent exposure of general practitioners to CPR and measures of advanced life support [25,26,28,29]. Moreover, we observed lower chest compression rates in general practitioners. However, general practitioners did not differ from hospital physicians in the timeliness and amount of basic life support or in the number of leadership utterances.

It is noteworthy that the rating of one owns team performance did not correlate with objective performance measures. Moreover, hardly any of the participants recalled delays, interruptions and other significant shortcomings when asked about their experience at the beginning of the video-assisted debriefing. These results suggest that during CPR health-care workers do not realise deviations from algorithms and question the value of narratives of medical emergencies. To the best of our knowledge, there are no previous studies that compared the ratings of one owns performance during medical emergencies with objective data collected during the same events. However, systematic discrepancies between perceived and objective reality may have important implications for the practice of emergency medicine. Thus, this topic merits further investigations and medical simulation appears to be perfectly suited as research tool.

What are the clinical implications of our findings? Recent data demonstrate that delayed defibrillation is associated with lower rates of survival and worse neurological and functional outcomes [14]. A delay in defibrillation of 40 sec will increase mortality by approximately 5% [30]. Animal data demonstrate a reduced survival rate after frequent or prolonged interruptions of cardiac massage [18,19,31]. Thus, the combination of delayed defibrillation and reduced hands-on time is of high clinical relevance as the expected impact on mortality and neurological outcome is substantial. All physicians are potential first responders in medical emergencies. Thus, they should be aware that structuring one's own team during CPR is an important prerequisite for a timely and effective team performance. All physicians, but especially general practitioners should be encouraged to use a defibrillator as soon as one is available [25,28,29]. In addition, physicians should be aware that the process of team-building is of high relevance for the quality of medical treatment.

Limitations of simulator-based studies include realism of both scenario and behaviour of the participants. However, the perceived realism of our scenario was very high (median rating 9 on a scale with a maximum of 10) as was the perceived realism of the participants' own behaviour (median rating 8). Moreover, the behaviour of our participants during the simulation and during the debriefing indicated strong involvement. Thus, it is unlikely that our findings are significantly affected by a lack of realism and/or by participants taking the simulation not seriously. A further limitation of the present study is that the preformed teams were preformed only very shortly before the cardiac arrest. Thus, the difference to ad-hoc forming teams may be even greater if longer standing preformed teams were to be studied.

Some authors used trained observers, video camera recording, or defibrillators capable of recording chest compressions and ventilation to evaluate the performance during real CPR [8,10,17]. However, ensuring the presence of trained observers at the very onset of a cardiac arrest is very difficult to achieve. Likewise, recording equipment is usually made functional during and not prior to resuscitation. Thus, both observers and recording equipment usually miss the performance during the initial phase of a cardiac arrest. A particular strength of our simulator-based study is thus the recording of objective data from both "patient" and participants right from the start of the cardiac arrest. Further strengths include a comparatively high number of participants, a controlled intervention applied in a randomized fashion, and identical conditions for all participants. Thus, in the present study medical simulation allowed the investigation of issues that for a variety of medical, practical, and ethical reasons are impossible to investigate in real patients.

## Conclusion

Hands-on time and time to defibrillation, two performance markers of CPR with a proven relevance for medical outcome, are significantly and negatively affected by shortcomings in the process of ad-hoc team-building and particularly deficits in leadership. Team-building has thus to be regarded as an additional task imposed on teams forming ad-hoc during CPR with a substantial impact on outcome. All physicians should be aware that structuring one's own team during CPR is a prerequisite for a timely and effective performance of life-saving measures. Future research should assess how physicians can improve their team-building abilities. Moreover, future guidelines and training in CPR should address the process of team-building.

## Competing interests

To ensure its economic survival, the simulator centre at the University of Basel offers educational workshops for physicians. In order to separate marketing activities from educational and research activities, the marketing of the workshops has been outsourced to Didavis AG, a company owned by one of the authors (RZ). However, the authorship of RZ is exclusively due to his academic contributions to the present study. Physicians taking part in our workshops can either subscribe individually or can be invited by companies using educational grants to subscribe for complete workshops or parts thereof. Physicians subscribing individually may, on their private initiative, be completely or partly sponsored by an educational grant of a third party. As a general rule, no third party, and especially no sponsoring company, is involved in any aspect of the research activities of the simulator centre at the University of Basel. Thus, the authors certify that no third party has been involved in any aspect of the present study.

Because the simulator centre at the University of Basel could not exist without the income generated by educational workshops, all authors had an interest that such workshops could be conducted in the past and have a continuing interest that workshops can be conducted in the future. Beyond that the authors declare that they have no competing interests.

## Authors' contributions

SH participated in data collection, data analysis, data interpretation and helped to draft the manuscript; FT participated in obtaining funding, the study design, data collection, data analysis, and data interpretation; NKS participated in obtaining funding, the study design, and data interpretation; RZ participated in the study design and data collection; MS participated in the study design and data collection; MB participated in the study design and data collection; PRH participated in the study design and data interpretation; SM participated in obtaining funding, the study design, data collection, data analysis, data interpretation and drafted the manuscript. All authors read and approved the final version of the manuscript.

## Pre-publication history

The pre-publication history for this paper can be accessed here:



## Supplementary Material

Additional file 1 **The Consort Flowchart.** The Graph provided shows the Consort flowchart of the study. Click here for file
