# Supplementary material for: Hands-on time during cardiopulmonary resuscitation is affected by the process of teambuilding: a prospective randomised simulator-based trial
Source: BMC Emerg Med. 2009 Feb 14;9:3. doi: 10.1186/1471-227X-9-3 (PMC2656452; doi:10.1186/1471-227X-9-3)
Supplement: Additional file 1 — The Consort Flowchart. The Graph provided shows the Consort flowchart of the study. [file 1471-227X-9-3-S1.doc]

Assessed for eligibility (n = 100)

Excluded (n = 0)

Not meeting inclusion criteria

(n = 0)

Refused to participate

(n = 0)

Other reasons

(n = 0)

Allocation

Analysis

Follow-Up

Enrollment

Analyzed (n = 50)

Excluded from analysis (n = 0)

Lost to follow-up (n = 0)

Discontinued intervention

(n = 0)

Allocated to intervention **ad-hoc**

(n = 50)

Received allocated intervention

(n = 50)

Did not receive allocated intervention

(n = 0)

Lost to follow-up (n = 0)

Discontinued intervention

(n = 0)

Allocated to intervention **preformed**

(n = 50)

Received allocated intervention

(n = 50)

Did not receive allocated intervention

(n = 0)

Analyzed (n = 49)

Excluded from analysis (n = 1)

Reason: **incomplete video recording**

**Randomization**
